# Human photoreceptor cells from different macular subregions have distinct transcriptional profiles

**DOI:** 10.1093/hmg/ddab140

**Published:** 2021-05-20

**Authors:** Andrew P Voigt, Nathaniel K Mullin, S Scott Whitmore, Adam P DeLuca, Erin R Burnight, Xiuying Liu, Budd A Tucker, Todd E Scheetz, Edwin M Stone, Robert F Mullins

**Affiliations:** Department of Ophthalmology and Visual Sciences, the University of Iowa Carver College of Medicine, Iowa City, IA 52242, USA; Institute for Vision Research, the University of Iowa, Iowa City, IA 52242, USA; Department of Ophthalmology and Visual Sciences, the University of Iowa Carver College of Medicine, Iowa City, IA 52242, USA; Institute for Vision Research, the University of Iowa, Iowa City, IA 52242, USA; Department of Ophthalmology and Visual Sciences, the University of Iowa Carver College of Medicine, Iowa City, IA 52242, USA; Institute for Vision Research, the University of Iowa, Iowa City, IA 52242, USA; Department of Ophthalmology and Visual Sciences, the University of Iowa Carver College of Medicine, Iowa City, IA 52242, USA; Institute for Vision Research, the University of Iowa, Iowa City, IA 52242, USA; Department of Ophthalmology and Visual Sciences, the University of Iowa Carver College of Medicine, Iowa City, IA 52242, USA; Institute for Vision Research, the University of Iowa, Iowa City, IA 52242, USA; Department of Ophthalmology and Visual Sciences, the University of Iowa Carver College of Medicine, Iowa City, IA 52242, USA; Institute for Vision Research, the University of Iowa, Iowa City, IA 52242, USA; Department of Ophthalmology and Visual Sciences, the University of Iowa Carver College of Medicine, Iowa City, IA 52242, USA; Institute for Vision Research, the University of Iowa, Iowa City, IA 52242, USA; Department of Ophthalmology and Visual Sciences, the University of Iowa Carver College of Medicine, Iowa City, IA 52242, USA; Institute for Vision Research, the University of Iowa, Iowa City, IA 52242, USA; Department of Ophthalmology and Visual Sciences, the University of Iowa Carver College of Medicine, Iowa City, IA 52242, USA; Institute for Vision Research, the University of Iowa, Iowa City, IA 52242, USA; Department of Ophthalmology and Visual Sciences, the University of Iowa Carver College of Medicine, Iowa City, IA 52242, USA; Institute for Vision Research, the University of Iowa, Iowa City, IA 52242, USA

## Abstract

The human neural retina is a light sensitive tissue with remarkable spatial and cellular organization. Compared with the periphery, the central retina contains more densely packed cone photoreceptor cells with unique morphologies and synaptic wiring. Some regions of the central retina exhibit selective degeneration or preservation in response to retinal disease and the basis for this variation is unknown. In this study, we used both bulk and single-cell RNA sequencing to compare gene expression within concentric regions of the central retina. We identified unique gene expression patterns of foveal cone photoreceptor cells, including many foveal-enriched transcription factors. In addition, we found that the genes *RORB1, PPFIA1* and *KCNAB2* are differentially spliced in the foveal, parafoveal and macular regions. These results provide a highly detailed spatial characterization of the retinal transcriptome and highlight unique molecular features of different retinal regions.

## Graphical abstract

**Figure:**
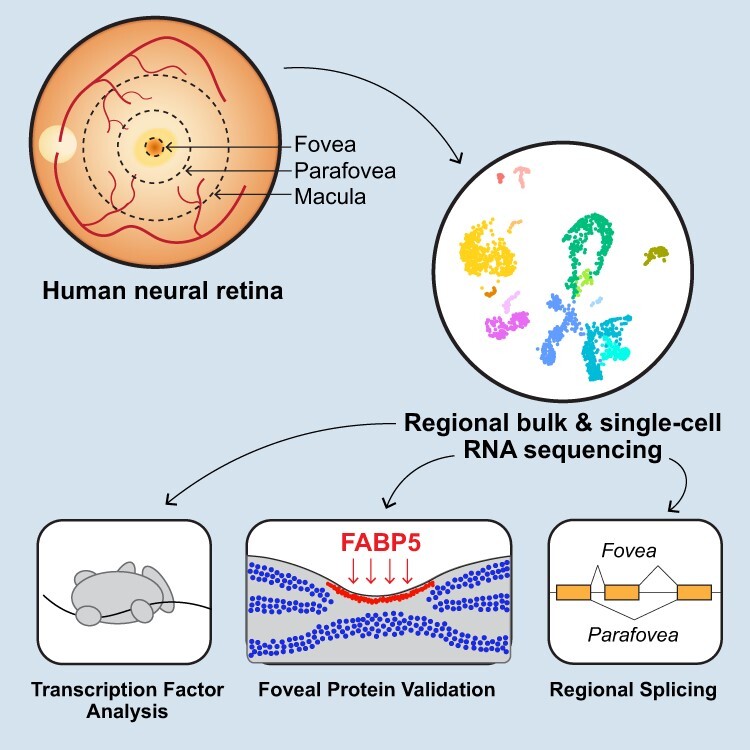

Retinal diseases can selectively affect some regions and spare others. Voigt *et al.* used single-cell RNA sequencing to identify gene expression differences within concentric regions of the human retina. Within the fovea, they identify region-specific transcription factors, gene splicing and protein expression.

## Introduction

The human retina shows a high degree of topographic heterogeneity. The central 6 mm of the human retina is referred to as the macula, which is clinically recognizable as the area between the superotemporal and inferotemporal vascular arcades. The macula may be broadly divided into four concentric zones with unique anatomic and physiologic features (1). The central most region of the macula is called the *foveola*, which is ~250–300 μm in diameter and consists exclusively of medium and long wavelength cone photoreceptor cells and Müller glia. Bipolar cells and retinal ganglion cells (RGCs) are radially displaced away from the foveola, creating a pit of tightly packed cone photoreceptor cells and Müller cell processes (2). Extending beyond the foveola is the *fovea*, which is ~1.85 mm in diameter and corresponds to the central 5.5° of the visual field. The fovea contains all inner retinal cell types, and rod and cone photoreceptor cells reach approximately equal densities within this region (1). The *parafovea* is ~3 mm in diameter and contains an abundance of RGCs bodies that were displaced from the foveola and fovea. Finally, the *perifovea* is ~6 mm in diameter. The perifovea contains a decreasing density of RGCs and more abundant rod photoreceptor cells.

Cells within the foveolar retina have unique anatomic and functional properties. For example, foveolar (medium or long wavelength) cone photoreceptors have long and thin outer segments as well as narrow, non-tapering inner segments and extended axons. Each cone photoreceptor synapses with one midget ON and one midget OFF bipolar cell, and each midget bipolar cell synapses with a single corresponding ON/OFF midget RGC (3,4). As the distance from the foveola increases, cone photoreceptors become gradually shorter and have wider inner segments (1). More peripheral cones have cone-shaped inner segments and shorter outer segments: the classical cone morphology described by Cajal (5). Multiple peripheral cone photoreceptors provide synaptic input to a single bipolar cell, and peripheral RGCs have even larger dendritic trees that receive input from multiple bipolar cells. Collectively, the tight packing of foveolar cone photoreceptor cells and their one-to-one synapses with bipolar cells and ganglion cells provides exceptionally high acuity vision from this very small retinal region.

**Figure 1:**
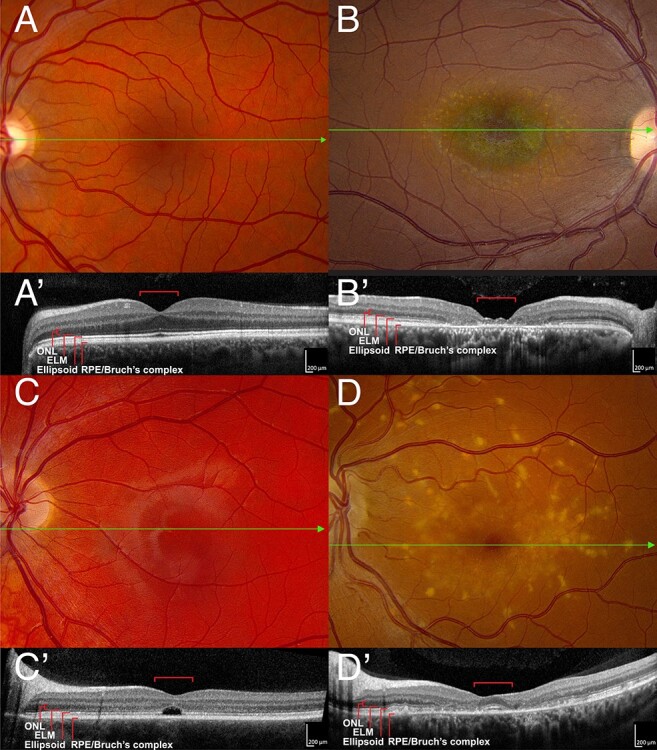
ABCA4-associated bullseye maculopathy (**A**). A normal color fundus photograph of the left eye of a 45-year-old man with 20/20 acuity. The green line shows the position of the OCT B-scan shown in (**A**′). In the OCT scan, the red brackets span 1 mm and are centered on the fovea. (**B**) Color fundus photograph of the right eye of a 30-year-old man with ABCA4-associated Stargardt disease and 20/80 acuity. The RPE and choriocapillaris are near normal in the central 500 μm giving rise to the dark-red color. The RPE and choriocapillaris are lost in the parafovea revealing the blue green color of the normal choroidal pigment. More anteriorly, yellowish collections of lipofuscin (flecks) can be seen at the level of the RPE. The green line shows the position of the OCT B-scan shown in (**B′**). There is some persistence of photoreceptors in the central 500 μm but complete loss of the outer retina and RPE in the parafovea. (**C**) Color fundus photograph of the left eye of an 18-year-old man with ABCA4-associated Stargardt disease and 20/200 acuity. The green line shows the position of the OCT B-scan shown in (**C**′). There is a selective loss of photoreceptors in the central 500 μm. The external limiting membrane appears to be intact. (**D**) Color fundus photograph of the left eye of a 42-year-old woman with ABCA4-associated Stargardt disease and 20/20 acuity. The foveola is preserved. Coarse yellow flecks are present throughout the macula. The green line shows the position of the OCT B-scan shown in (**D′**). The ellipsoid zone and external limiting membrane are clearly visible in the central 500 μm. The latter structures are lost in the parafovea but are present and normal in the more anterior retina. The flecks lie at the level of the RPE.

Given the quite dramatic differences in the cellular composition and physiologic function of different regions of the retina, it is perhaps not surprising that some disease processes tend to selectively affect some regions and spare others. For example, age-related macular degeneration is much more likely to injure structures in the central 6 mm of the retina. In contrast, most forms of retinitis pigmentosa tend to affect the more peripheral retina more severely and earlier than the macula. Some disorders, such as *ABCA4*-associated Stargardt disease, can even affect the foveola, parafovea and perifovea in a differential manner giving rise to a ‘bullseye’ appearance in the macula (Fig. 1B and B′). This differential effect varies from patient to patient, suggesting that the behavior of other genes in the genetic background can modify the impact of *ABCA4* mutations on the macular photoreceptors. Some individuals experience selective loss of their foveola early in their disease (Fig. 1C and C′), whereas others retain their foveola as they lose more anterior photoreceptors (Fig. 1D and D′).

Gene expression studies may provide insight into the functional differences between these retinal regions. Previous RNA sequencing experiments have identified molecular differences between the central versus peripheral retina at both the bulk (6) and single cell (7–11) levels. In this study, we sought to extend these findings by comparing gene expression between the fovea and directly surrounding parafoveal retina. Using eight human donor retinas, we performed complementary bulk (*n* = 4) and single-cell (*n* = 4) RNA sequencing experiments across paired foveal (1 mm) and parafoveal (4 mm) samples. We identified differentially spliced genes between the fovea and parafovea as well as numerous expression differences between these central regions of human retina.

## Results

### Bulk RNA sequencing of foveal, parafoveal and macular retina

**Figure 2:**
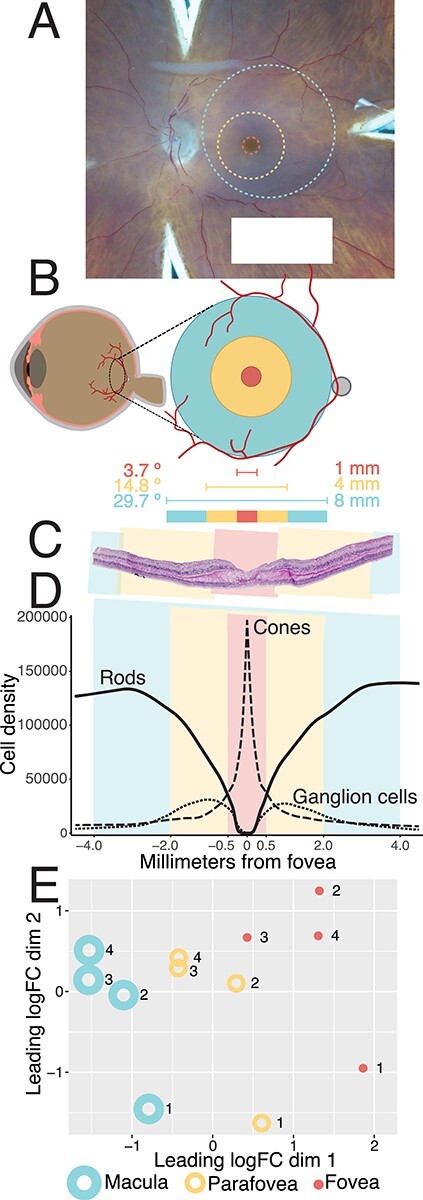
Comparing gene expression in foveal (1 mm), parafoveal (4 mm) and macular (8 mm) retina. (**A**) A hypothetical 1 mm foveal-centered punch (red), 4 mm parafoveal punch (yellow), and 8 mm macular punch (blue) are overlayed on a gross photo of a human donor eye. Punches were acquired concentrically; however, the position of the 8 mm macular punch was slightly adjusted on a case-by-case basis to avoid the optic nerve head. (**B**) The contribution of each punch to the visual field was estimated (12). (**C**) Hematoxylin and eosin histological staining of fovea-centered retina. A perfectly centered 1 mm foveal punch (red) includes the entire foveal pit. (**D**) Density of cones, rods and RGCs across each of the investigated regions of macular retina (14). Cones reach their peak density within the foveola, which is completely captured in a perfectly centered 1 mm punch. (**E**) After bulk RNA-sequencing of four human donors, multidimensional scaling reveals separation of macular, parafoveal, and foveal retinal samples, suggesting that each region has a unique transcriptome.

Different regions of the human macular retina are susceptible to selective degeneration or preservation in different retinal diseases. In order to better study the regional topography of this important retinal region, we acquired three distinct trephine punch biopsies of macular retina for bulk RNA sequencing. From four human donors (Table 1), we acquired concentric trephine punches consisting of a 1 mm foveal-centered punch, a 4 mm parafoveal punch and an 8 mm macular punch of retinal tissue (Fig. 2A). The first two punches were centered on the fovea while the position of the 8 mm punch was adjusted on a donor-by-donor basis to avoid inclusion of the optic nerve head. Next, the contribution of each punch to the visual field was estimated [(12); Fig. 2B]. The 1 mm foveal punch corresponds to the central 3.7° of vision, whereas the macular punch extends to roughly 30 visual degrees. As a reference, the size of the foveal and parafoveal punches was compared with a hematoxylin and eosin-stained histological retinal section (Fig. 2C). The average diameter of the adult foveal depression is 0.65–0.7 mm (13) and is expected to be included in a perfectly centered 1 mm foveal-centered punch.

**Table 1:** Donor information

Donor	Experiment	Age	Sex	PMI	Eye	Cause of death	Ophthalmologic history
Donor 1	Bulk RNA-seq	60	M	7:35	OD	Multiple myeloma	N/A, histology WNL
Donor 2	Bulk RNA-seq	82	M	6:27	OS	Biliary obstruction	N/A, gross appearance and histology of OD WNL
Donor 3	Bulk RNA-seq	62	M	6:05	OS	Ischemic bowel	N/A, gross appearance and histology WNL
Donor 4	Bulk RNA-seq	71	M	5:46	OS	Lung cancer	N/A, gross appearance and histology WNL
Donor 5	scRNA-seq	59	M	6:59	OD	Perforated diverticulitis	Cataract surgery
Donor 6	scRNA-seq	63	F	6:47	OS	Lung cancer	N/A, gross appearance WNL
Donor 7	scRNA-seq	78	M	6:42	OS	Chronic respiratory failure	Extensive soft drusen; preserved foveal pigment
Donor 8	scRNA-seq	71	M	7:56	OS	Cardiogenic shock	N/A, gross appearance WNL

Demographic information for the eight human donor retinas utilized in these experiments. A total of four unique human donors were utilized for each the bulk RNA sequencing and single-cell RNA sequencing experiments. The ophthalmologic history and histology of the contralateral eye were examined for each donor. PMI, post-mortem interval; OD, oculus dexter (right eye); OS, oculus sinister (left eye); N/A, clinical information not available; WNL, within normal limits.

Different regions of neural retina have different cell type compositions as elegantly outlined in morphometric experiments by Curcio *et al.* (14,15). Our 1 mm punch completely encompasses the foveola, an avascular area of tightly packed cone photoreceptor cells where rod photoreceptor cells are absent (Fig. 2D). RGCs are radially displaced away from the fovea and reach their peak density within our 4 mm parafoveal punch. Likewise, rod photoreceptor cells are absent from the center of the fovea but gradually increase in density with increasing eccentricity.

We performed bulk RNA sequencing on the isolated punches of foveal, parafoveal and macular retina. After mapping reads to the genome, we compared the expression profiles of these three retinal regions. The three regions exhibited well-separated global transcriptomes after applying the dimensionality reduction technique of multidimensional scaling [(16); Fig. 2E]. To identify genes enriched in each retinal region, we performed differential expression analysis between each region pair (fovea-parafovea, fovea-macula and parafovea-macula; Supplementary Material, Table S1). A total of 359 genes were differentially expressed [abs(logFC) > 1, FDR < 0.01] between the fovea and the parafovea, which were the two most transcriptionally similar retinal regions (Supplementary Material, Fig. S1A and B). In contrast, 1667 genes were differentially expressed between the fovea and the macula, which were unsurprisingly the most dissimilar retinal regions. Next, we identified functional categories of genes enriched in the fovea using WebGestalt [(17); Supplementary Material, Fig. S1C]. Genes enriched in the fovea were involved in phototransduction, mineral absorption, GABAergic signaling and neuroactive ligand–receptor interactions pathways.

Because of the limitations of bulk RNA sequencing, it is possible that the observed regional expression differences could simply be due to the different cell composition of each retinal region (Fig. 2D, Supplementary Material, Fig. S1D). For example, the rod-specific opsin gene *RHO* was significantly enriched in the parafoveal retina versus the foveal retina. This parafoveal enrichment has two possible explanations. First, it is possible that the rods surrounding the fovea (some of which are included in a 1 mm foveal punch) have less *RHO* expression than parafoveal rods. More likely, it is possible that the rods in the 1 and 4 mm punches have comparable *RHO* expression, but that the increased proportion of parafoveal rods (Fig. 2D) results in higher detection of rod-specific genes in this region.

### Foveal versus parafoveal single-cell RNA sequencing

**Figure 3:**
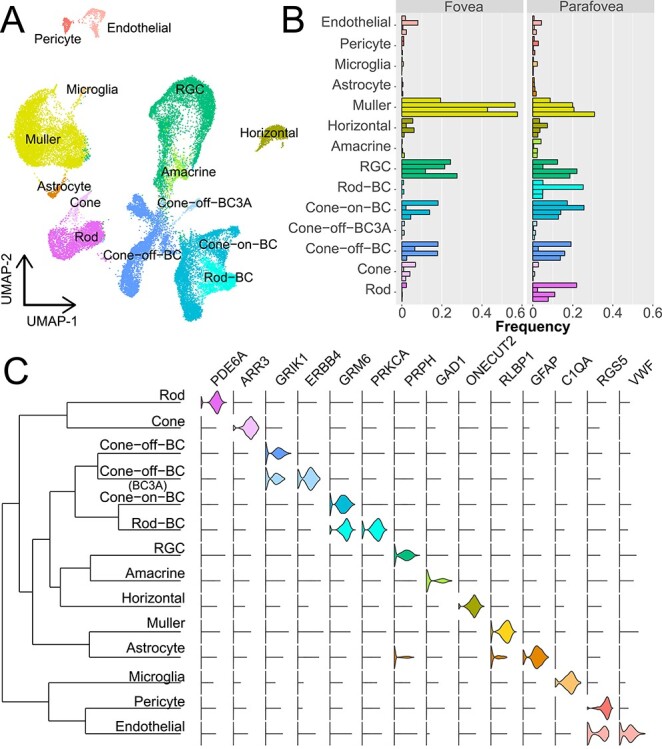
scRNA-seq of foveal and parafoveal retina. (**A**) scRNA-seq of paired foveal and parafoveal retina samples from four human donors. After computational processing, uniform manifold approximation and projection dimensionality reduction was performed to visualize clusters of cells. A total of 5856 foveal and 28 637 parafoveal cells were recovered after filtering. (**B**) The proportion of recovered cells in the fovea and parafovea were calculated for each donor. Each individual bar represents the proportion of a recovered cell type in one donor. Note that in the fovea, cone photoreceptor cells are more abundant than rod photoreceptor cells, but this pattern is reversed in the parafovea. (**C**) A dendrogram shows the relationships between each cluster of cells (left). Violin plots (right) depict the expression of previously reported cell-specific genes across each cluster.

To address this limitation, we performed a complementary single-cell RNA sequencing (scRNA-seq) experiment to compare gene expression in paired foveal versus parafoveal retina across four independent human donors (Table 1). After mapping the reads and filtering low quality cells, we recovered 5856 foveal and 28 637 parafoveal cells that corresponded to all major retinal populations (Fig. 3A and C). First, we compared the proportion of recovered foveal versus parafoveal cells across each retinal cell class (Fig. 3B). Based on the previously published distributions of retinal cell types (14,15), we expected that our 1 mm foveal punch would contain a ratio of 1 cone photoreceptor to 0.5 rod photoreceptors to 0.3 RGCs. Although this mirrored the observed proportion of foveal photoreceptors (1 cone: 0.2 rods), more RGCs were recovered than anticipated (1 cone: 6.2 RGCs). In contrast, we predicted that our 4 mm parafoveal punch would contain 1 cone photoreceptor to 6.7 rod photoreceptors to 1.5 RGCs. Again, these predicted distributions approximated the observed ratio of parafoveal photoreceptors (1 cone: 10.3 rods), but RGCs were again recovered at a higher-than-predicted frequency (1 cone: 13.9 RGCs). Interestingly, Müller cells were the most abundantly recovered cell type in both the fovea (37% of all cells) and parafovea (22% of all cells).

#### Foveal versus parafoveal gene expression in cone photoreceptors

**Figure 4:**
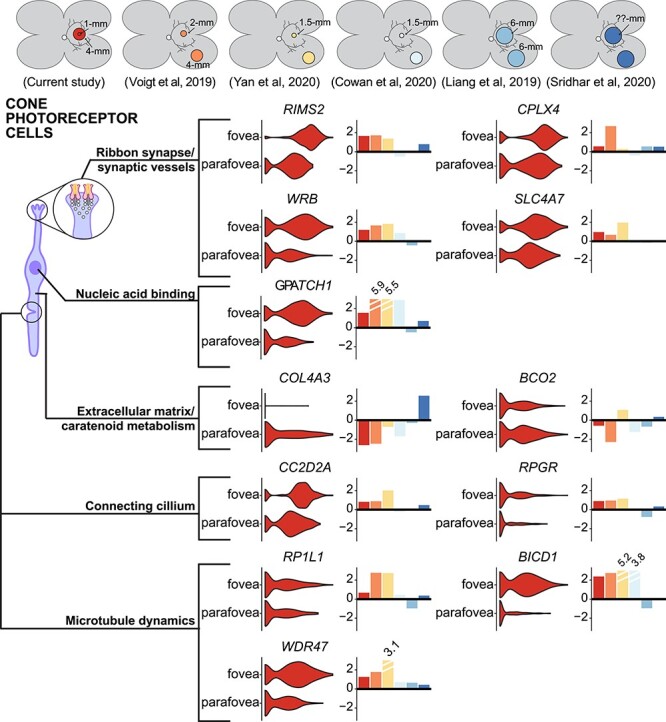
Gene expression in foveal versus parafoveal cone photoreceptor cells. A subset of differentially expressed genes between foveal and parafoveal cone photoreceptor cells are displayed. For each gene, a violin plot depicts the distribution of expression in the fovea and the parafovea. To the right of each violin plot, a barplot depicts the central (positive values) or peripheral (negative values) log-fold change enrichment of each gene across this study (red) and five independent regional retina scRNA-seq investigations (colors in top bar).

After comparing the proportion of foveal versus parafoveal cells, we next compared gene expression differences across these retinal regions (Supplementary Material, Table S2). First, we compared cone photoreceptor gene expression between the fovea and parafovea (Fig. 4). The most foveal enriched cone photoreceptor gene was *RIMS2*, a regulator of synaptic membrane exocytosis that has been implicated in a congenital cone-rod synaptic disorder (18). Several additional genes implicated in synaptic transmission were enriched in foveal cone photoreceptors, likely reflecting the unique synaptic characteristics of foveal cones. Such foveal enriched genes include *CPLX4*—which regulates synaptic vesicle fusion at the ribbon synapse (19), *WRB*—which supports ribbon synapse structure and function (20) and *SLC4A7*—which encodes a sodium bicarbonate transporter that regulates pH for proper sensory transmission (21). We compared the foveal enrichment of each of these genes with five independent scRNA-seq studies that explored gene expression between different retinal regions (7–11). Similar to the current study, *RIMS2*, *CPLX4*, *WRB* and *SLC4A7* were enriched in the central-most libraries across these five complementary investigations (Fig. 4).

In contrast, parafoveal cones were enriched in several genes compared with the fovea. In concordance with our previous studies (10), the carotenoid cleaving enzyme *BCO2* was more highly expressed in parafoveal cones. As BCO2 cleaves xanthophyllic substrates such as lutein (22), the lack of foveal *BCO2* expression may contribute to the accumulation of foveal pigments. In addition, foveal cones lacked expression of the collagen alpha3(IV) subunit gene *COL4A3*. Interestingly, single-nucleotide polymorphisms in *COL4A3* have been significantly associated with age-related macular degeneration (23). Parafoveal cones express more *COL4A3* than any other retinal cell type or any cell type in the RPE/choroid [after comparison with single-cell data from (24)]. Each of these regional enrichments was reproducible in the majority of the five independent scRNA-seq studies (Fig. 4).

**Figure 5:**
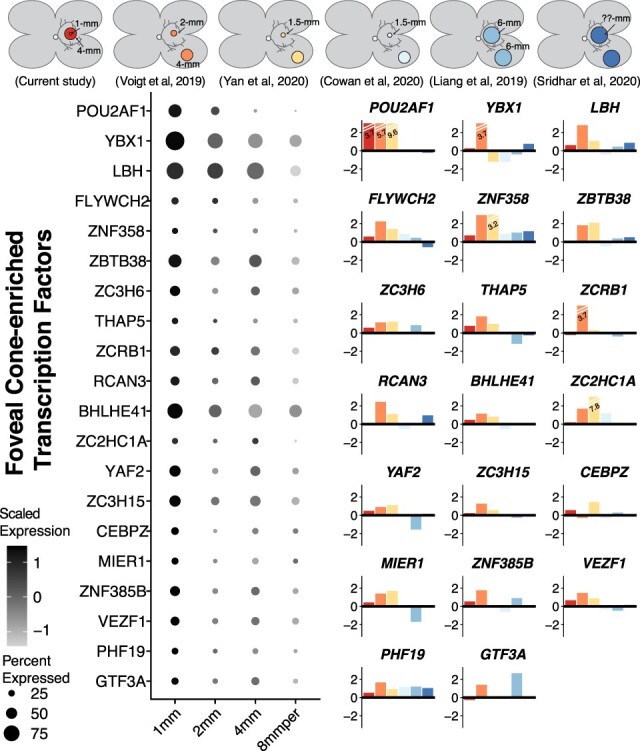
Transcription factors enriched in foveal cones (**A**). A subset of differentially expressed transcription factors enriched in foveal cone photoreceptor cells are displayed. Data from the current study was combined with our identically-processed foveal and peripheral retina samples from previous investigations (10,26). For each gene, a dotplot displays the relative expression level of each transcription factor across five retinal regions, with dark colors corresponding to high expression. The size of each circle is proportional to the percent of cone photoreceptors that express the transcription factor. (**B**) A barplot depicts the central (positive values) or peripheral (negative values) log-fold change enrichment of each gene across this study (red) and five independent regional retina scRNA-seq investigations (colors in top bar).

Foveal cone photoreceptor cells have unique morphologic and electrophysiologic features (1). Therefore, we set out to identify region-specific transcription factors that may contribute to these distinct characteristics. After identifying fovea-enriched genes, we used the systematic gene ontology database (SysGO) to highlight region-specific transcription factors [(25); Supplementary Material, Table S3]. As cone photoreceptor morphology changes gradually with increasing distance from the foveola, we compared the expression characteristics of these transcription factors across several central and peripheral retinal regions from our previously published studies [Fig. 5; (10,26)]. In addition, the central enrichment of each transcription factor was compared across five independent datasets as described above (Fig. 5). Compared with the parafovea, foveal cones showed increased expression of the transcription factors *POUF2A1*, *YBX1* and *LBH*. A similar analysis was performed to identify peripherally enriched transcription factors in cone photoreceptors (Supplementary Material, Fig. S3).

#### Foveal versus parafoveal gene expression in rod photoreceptors and Müller cells

**Figure 6:**
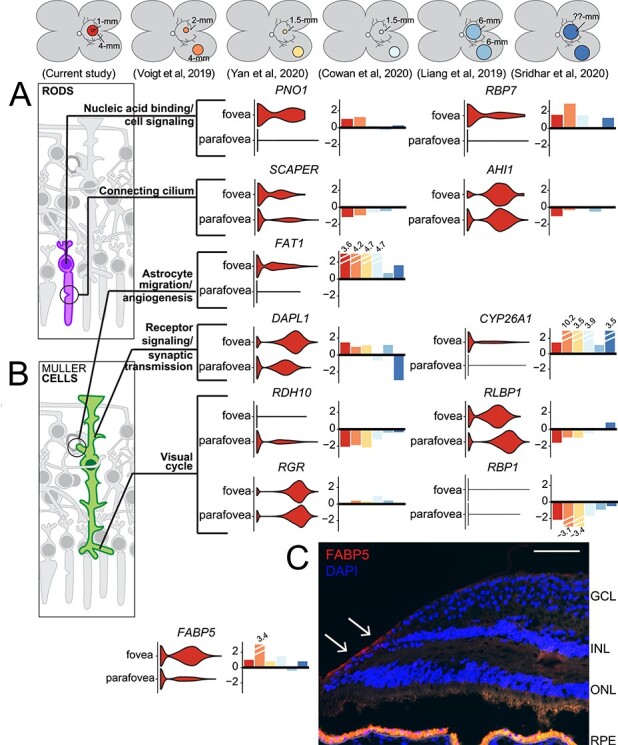
Gene expression in foveal versus parafoveal rod photoreceptor cells and Müller cells. A subset of differentially expressed genes between foveal and parafoveal (**A**) rod photoreceptor cells and (**B**) Müller cells are displayed. For each gene, a violin plot depicts the distribution of expression in the fovea and the parafovea. To the right of each violin plot, a barplot depicts the foveal (positive values) or parafoveal (negative values) log-fold change enrichment of each gene across this study (red) and five independent regional retina scRNA-seq investigations (colors in top bar). (**C**) Immunohistochemical localization of FABP5 in the foveal retina. Scale bar = 100 micrometer.

Next, we compared foveal versus parafoveal gene expression in rod photoreceptor cells (Fig. 6A). Although rods are depleted from the foveal center, the 1 mm punch is still expected to contain almost 500 000 rods [(14); Fig. 2]. Foveal rod photoreceptors were enriched in the RNA-binding gene *PNO1*, which is required for ribosome assembly (27) and the retinol binding protein *RBP7.* In contrast, parafoveal rods were enriched for several cilia-localized genes including *SCAPER*, which has been implicated in Bardet-Biedl syndrome (28), and *AHI1*, which is a mendelian cause and modifier of Joubert syndrome (29). Each of these regional enrichments was consistent across the majority of other regional scRNA-seq studies.

We also observed regional differences in Müller cell gene expression (Fig. 6B). Foveal Müller cells were enriched for *DAPL1*, a G-coupled protein receptor that contains a single-nucleotide polymorphism associated with AMD (30). Likewise, foveal Müller cells were enriched for *FAT1*, a protocadherin needed for retinal vascular organization (31) and *CYP26A1*, a retinoic acid catabolizing enzyme (32,33). Finally, foveal Müller cells were enriched for *FABP5*, which encodes a fatty-acid binding protein that can regulate retinoic acid signaling (34). We validated the protein-level expression of FABP5 with immunohistochemistry and demonstrated the specific localization of this protein along the internal limiting membrane of the fovea of three independent donors (Fig. 6C).

Müller glia cells have an important role in the visual cycle and can help re-isomerize visual opsins in cone photoreceptor cells (35). We observed that Müller cells strongly and specifically expressed the visual cycle genes *RDH10*, *RGR*, *RLBP1* and *RBP1.* Although each of these transcripts was expressed by both foveal and parafoveal Müller cells, we observed a slight parafoveal enrichment of *RDH10*, *RLBP1* and *RBP1* expression (Fig. 6B).

### Alternate isoform expression in different retinal regions

Many popular scRNA-seq platforms limit sequencing to the 3′ region of each gene. Although these library construction strategies allow for gene expression investigation across thousands of independent cells, they unfortunately preclude analyzing the relative expression of most isoforms. In contrast, bulk RNA-sequencing pools RNA contributions from different cell types, but the sequencing is not biased towards the 3′ end of each gene, resulting in full-length coverage of RNA transcripts. To combine the strengths of these technologies, we used the bulk RNA-sequencing data to identify alternatively spliced genes between the foveal, parafoveal and macular retina. We then used the scRNA-seq data to pinpoint which cell type maximally expressed each of these alternatively spliced genes.

**Figure 7:**
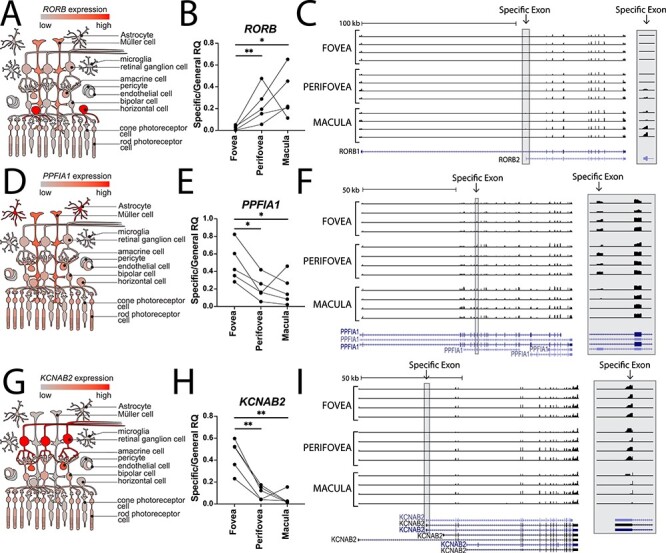
Regionally expressed retinal isoforms. Using the bulk RNA sequencing data, *RORB* (**A–C**), *PPFIA1* (**D–F**) and *KCNAB2* (**G–I**) were identified as differentially spliced between the fovea, parafovea and macula. The scRNA-seq data was used to identify which cell type most highly expressed each gene (left column). Differential isoform abundance in each region was validated by RT-qPCR isoform-specific primers (middle column, see Materials and methods). Coverage tracks show reads mapped to each exon (right column), with an inset to illustrate the differentially expressed exons at higher magnification (grey box).

We identified three different genes with regionally enriched isoforms. First, the retinoid-related orphan receptor B (*RORB*) gene was enriched in the macular and parafoveal retina and depleted in the fovea (exon 1, isoform ID NM_001 365 023, hg19 Chr9:77 230 450–77 230 555, Fig. 7A–C). *RORB* demonstrated the highest expression in horizontal cells and Müller cells (Fig. 7A), with comparable expression between the fovea and parafovea (Supplementary Material, Table S2). Previous studies have shown that retinal *RORB* expression regulates circadian timing (36) and is important for neuronal differentiation and patterning (37,38). *RORB* encodes two isoforms with distinct N-terminal domains. The *RORB1* isoform is required for differentiation of horizontal cells and amacrine cells (39). In contrast, the *RORB2* isoform induces *NRL* expression, driving the development of rod photoreceptors (40). In the bulk RNA sequencing data, the *RORB2*-specific exon was expressed at higher levels within the macula and parafovea than the fovea (Fig. 7C), and this regional expression was validated with qPCR with three of the original RNA-seq libraries and two independent donors (Fig. 7B). Despite low expression in rod photoreceptor cells, it is possible that the macular enrichment of the *RORB2* isoform is driven by the increased proportion of rods in this retinal region.

We also detected regionally enriched isoforms for the gene *PPFIA1* (exon 10, isoform ID NM_001 378 006, hg19 Chr11:70 179 128–70 179 202, Fig. 7D–F), which encodes liprinα1, a regulator of synapse organization and development (41). This gene was most highly expressed in astrocytes and Müller glia (Fig. 7D). Using the bulk RNA-seq data, we detected a foveal and parafoveal enriched exon (Fig. 7F), and this regional enrichment was validated with qPCR (Fig. 7E). Similarly, we detected regionally enriched isoforms for the gene *KCNAB2* (exon 1, isoform ID NM_172 130, hg19 Chr1:6 086 393–6 086 505, Fig. 7G–I). *KCNAB2* encodes the voltage-gated potassium channel β2 subunit (42), which demonstrated the highest expression in RGCs and amacrine cells (Fig. 7G). Alternative splicing of *KCNAB2* results in many transcript variants, and an exon unique to several of these isoforms was enriched in the fovea and parafovea compared with the macula (Fig. 7I). This exon also demonstrated foveal enrichment by qPCR analysis of RNA from three of the RNA-seq donors as well as two independent donors (Fig. 7H).

### Interactive exploration of regional expression differences

We recently developed Spectacle, a free online resource for interactive analysis of ocular scRNA-seq data (43) (available at https://singlecell-eye.org). We have uploaded scRNA-seq data from the current investigation to Spectacle, making this dataset available for querying gene expression, reclustering, and highly flexible differential expression analysis. As regional retinal gene expression is of particularly high interest (7–11), we also developed a new way to interactively compare retinal gene expression across five different retinal studies. The new regional expression tool allows users to determine whether a gene of interest is enriched centrally or peripherally across six independent datasets, enabling the user to generate plots such as those in Figures 4 and 6 for any gene in any cell type. Publication quality results are easily downloadable in both graphical and tabular formats.

## Discussion

Although the photoreceptors and Müller cells of different retinal regions are superficially similar, inherited degenerative diseases of the retina often impact distinct anatomical regions in strikingly different ways. As a result, improving the regional specificity of retinal gene expression is likely to improve our understanding of the pathophysiology of such region selective diseases (44). ScRNA-seq has many advantages in identifying unique expression features of the fovea. In our bulk RNA sequencing analysis of foveal versus parafoveal gene expression, we detected a total of 359 differentially expressed genes. There are two possible biological factors that explain why these genes were detected at different levels between these retinal regions. First, it is possible that one cell type expresses a gene at a higher level in either the fovea or parafovea. We regard this scenario as a ‘true quantitative expression difference’ detected by bulk RNA sequencing. Second, as different regions of the retina have different cellular compositions (Fig. 2D), one cell type could express the same amount of a gene in the fovea and the parafovea, but this cell type could also be much more abundant in one region than the other thereby contributing a greater fraction of the total transcripts. We regard this second scenario as a ‘cell composition difference’. Using the scRNA-seq data, we identified which cell type most highly expressed each of the 359 bulk RNA sequencing differentially expressed genes between the fovea and parafovea. Next, we determined if there was a true difference in foveal versus parafoveal expression in this maximally expressing cell type (defined by an absolute logFC > 0.5) using the scRNA-seq data. Only 15% of genes with differential expression in bulk studies were believably enriched in one retinal region based on these criteria. This suggests that the majority of the bulk RNA sequencing differentially expressed genes was identified due to cell composition differences between the fovea and parafovea.

Foveal cone photoreceptor cells mediate our highest acuity vision and thus these cells represent a promising target for autologous induced pluripotent stem cell replacement in the setting of retinal degeneration (45,46). Foveal cones have unique morphologies and synaptic connections, and it is possible that region-specific transcription factors drive and maintain these unique characteristics (Fig. 5). Compared with the parafovea, foveal cones showed increased expression of the transcription coactivator *POUF2A1,* which interacts with OCT1 and OCT2. Previous studies have demonstrated that POU2AF1 regulates B- and T-cell activation (47), and more recent studies have detected POU2AF1 activity in non-immune tissues such as the human airway epithelium (48). Likewise, foveal cones were enriched in the nucleic acid binding protein *YBX1*. YBX1 regulates the ribonucleoprotein complex PCR2, and this YBX1-mediated regulation spatially and temporally modulates neurodevelopmental gene expression (49). Foveal cones were also enriched in the limb bud and heart (LBH) transcriptional coactivator, which interacts with OTX2 to regulate photoreceptor differentiation in a zebrafish model (50). The identification of such transcription factors may guide generation of more foveal-like cone photoreceptor cells from retinal organoids.

In addition to cone photoreceptor cells, Müller glia are the only other cell type found within the foveola. Foveal Müller cells were enriched for *DAPL1*, a G-coupled protein receptor that contains a single-nucleotide polymorphism associated with AMD (30). Interestingly, the *DAPL1* risk allele has been associated with diminished expression of two *DAPL1* isoforms. However, the association of this polymorphism with AMD is only significant in females. In addition, foveal Müller cells were enriched in two regulators of retinoic acid signaling. First, *CYP26A1* is a retinoic acid catabolizing enzyme. Loss of retinoic acid is important in foveal patterning. In the chicken, *CYP26A1* is regionally restricted to the central retina when the high-acuity area is forming (33). Similarly, *CYP26A1* expression is enriched in macular Müller cells in the human retina at gestational week 20 (51). Second, retinoic acid signaling is modulated by FABP5 expression. In cells with low FABP5 concentration, retinoic acid activates the classical retinoic acid receptor. In contrast, retinoic acid targets PPARB/G in cells with high FABP5 (34). The high foveal expression of FABP5 by Müller glia may modulate the effects of this important signaling molecule.

Several previous investigations have employed scRNA-seq to differentiate gene expression in the central versus peripheral human retina (7–11). In this study, we isolated cells from the smallest and the most specific foveal region described to date, and we compared cells from this region to the directly surrounding parafoveal retina. In addition, we completely reprocessed scRNA-seq data from five independent investigations. This reprocessing allowed us to assess the consistency of regional gene expression enrichment across different studies. However, it is worth noting that each of these experiments had unique tissue processing, dissociation methods, demographic criteria for human donors and sequencing strategies. For example, most of the studies we evaluated used scRNA-seq to profile gene expression, however Liang *et al.* profiled gene expression at the single-nucleus level. Likewise, Sridhar *et al.* compared central versus peripheral expression of fetal human retina, whereas all other studies we analyzed contained adult human donors. In addition, in the study by Yan *et al.*, peripheral rod photoreceptors were depleted with CD73-microbeads prior to sequencing, resulting in slightly different processing between the central and peripheral retinal cells. Interestingly, we observed slightly more study-to-study variation when comparing the peripherally enriched transcription factors in cone photoreceptor cells (Supplementary Material, Fig. S2). Overall, despite these differences in study design, the majority of regional expression differences found in this current study were reproducible across several independent datasets. To encourage similar comparisons, we have hosted all scRNA-seq data from this study on an interactive webserver at https://singlecell-eye.org (43). This webserver has a new regional expression functionality, which allows users to compare gene expression across multiple regional retina scRNA-seq datasets.

There are several limitations to this study. First, our recovered proportion of cells by scRNA-seq likely does not reflect the true cellularity of the retina. Although our ratio of cone-to-rod photoreceptor cells closely approximated previously described foveal cell counts (14), we recovered other retinal cell types such as Müller cells and RGCs at higher than anticipated rates (Fig. 3B). It is possible that the dissociation protocols selectively affect the viability of photoreceptor cells, which have a fragile inner segment-outer segment junction. Supporting this idea, we recovered a similar proportion of cone and rod photoreceptor cells in this experiment to other retina scRNA-seq studies that included a foveal sample (Supplementary Material, Fig. S2). Second, our scRNA-seq barcoding strategy limited sequencing to the 3′ region of each gene. Although this barcoding strategy increased the number of cells that we could recover in this experiment, it precluded analysis of splicing at the single-cell level. The bulk RNA-sequencing data allowed us to detect regionally expressed isoforms (Fig. 6), however alternative barcoding strategies would be required to map the expression of these isoforms to individual cells (52).

The expression profiles generated by this study reveal a specific foveal transcription signature and provide a rich resource for further study of this very important retinal region.

## Materials and Methods

### Experimental model and subject details

Human donor eyes were acquired from the Iowa Lions Eye Bank in accordance with the Declaration of Helsinki following consent of the donor’s next of kin. Tissue in this report was used in two transcriptomic studies (Table 1). Independent donors were acquired for bulk and scRNA-seq studies, and these samples have not been described previously.

### Bulk RNA-sequencing of foveal, parafoveal and macular human retina

Retinal tissue was isolated from donors 1–4 with 1, 4 and 8 mm trephine punch biopsies centered on the fovea. The overlying retinal tissue was separated from the underlying retinal pigment epithelium and choroid before flash freezing in liquid nitrogen. Frozen samples from donors 1–4 were removed from the freezer in parallel, and RNA was isolated immediately from each sample using the Single Cell RNA Purification Kit (Norgen, Ontario, Canada #51800) according to manufacturer’s instructions (protocol 1Bii initiating with step c). Briefly, punches were lysed in 350 ul Buffer RL + 3.5 μl BME. Based on cell number estimates in each punch (~1 × 10^6^ per macula punch, ~ 5 × 10^5^ per parafovea punch and ~1 × 10^5^ per fovea punch) amounts were scaled accordingly (350 μl × 5 per macula^*^, 350 μl × 2 per parafovea and 350 μl × 1 per fovea) and divided among the appropriate number of tubes before proceeding with the alcohol precipitation step (protocol 1Bii step d). On-column DNase treatment was performed using RQ1 DNase (10 μl per 100 μl total reaction volume each sample; Promega #M6101) for 30 min at room temperature. Total RNA was eluted in 12 μl UltraPure dH_2_O (ThermoFisher Scientific, Rockford, IL #10 977 023). Total RNA underwent 150 base pair paired-end sequencing on the Illumina HiSeq4000 platform (Illumina, San Diego CA).

### Computational analysis of bulk RNA-sequencing data

Sequenced reads were mapped to the human reference genome hg19 with STAR [v020201, (53)] and reads were quantified with featureCounts [Rsubread v1.28.1, (54)]. Differentially expressed genes were identified for each pairwise region comparison (e.g., fovea-parafovea, fovea-macula and parafovea-macula) with edgeR [v3.20.9, (16)]. Genes were determined to be differentially expressed if the absolute value of the log fold change was greater than one and the false discovery rate (FDR) was <0.01. Differential isoform abundance from the bulk RNA sequencing data was quantified with JunctionSeq (55).

### Real time PCR quantification of isoform abundance

RNA was isolated with the PicoPure RNA isolation kit (Applied Biosystems, Foster City CA) from 1, 4 and 8 mm retinal punches from *n* = 3 independent donors. cDNA was amplified with a high-capacity cDNA reverse transcription kit (Applied Biosystems, Foster City CA). Isoform abundance was assessed by RT-qPCR using SyBr green chemistry and isoform-specific primers. Primers were designed using the IDT PrimerQuest webtool. For each gene of interest, one primer set was designed to be specific to the regionally differentially expressed isoform (termed ‘specific’). Another primer set was designed to a region shared by both the differentially expressed isoform and all other isoforms of the gene (termed ‘general’). RT-qPCR reactions were prepared using Applied Biosystems PowerSYBR Green PCR Master Mix according to the manufacturer’s instructions and carried out using the QuantStudio 6 Flex instrument.

The relative abundance of transcripts was calculated using the ΔΔCt method. Ct values were first normalized to those of 18S, and then the difference of ΔCts for the ‘specific’ and ‘general’ primer sets for each gene and region was calculated. Relative quantity was calculated as 2^−ΔΔCt^. Data were plotted, and statistical analysis was performed using Prism 8. Ratio paired *t*-tests were performed between each pair of regions for each gene.

**Table:** 

Target	Forward primer	Reverse primer	Exons	Amplicon length (bp)
KCNAB2 specific	ATGTGTGACCTGGCGTTC	CCTCACTTATCCAGACTTGCTC	1 and 2	115
KCNAB2 general	GAACCTGGGCAAGTCTGG	GGTGATCTGGCCTCCGAA	4 and 5	76
RORB specific	ATGTGTGAGAACCAGCTCAAA	TAGTGGATCCCAGAGGACTTATC	1 and 2	98
RORB general	TAAGTCCTCTGGGATCCACTAC	GCCTTGGGCAGGAATAAGAA	2 and 3	101
PPFIA1 specific	CTCTGCTGCTAAGGAAGCTAAA	CTTCCATCTGTCGTAACCTTTCT	5 and 6	101
PPFIA1 general	AAGCAGTTAGAAGAAACACAACAC	CTTAGTCTCATGTGGTCTAGTTCAG	8 and 9	89
18S	CGGCTACCACATCCAAGGAAG	GCTGGAATTACCGCGGCTGCT	N/a	187

**Methods**  Table 1: qPCR primers for quantifying regional isoform abundance.

### Foveal and parafoveal retina scRNA-seq

Retinal tissue was isolated from donors 5–8 with 1 mm (foveal) and 4 mm (parafoveal) trephine punch biopsies centered on the fovea. To minimize inadvertently collecting cells from the retinal pigment epithelium or choroid, an 8 mm retinal punch was first collected and moved to a separate dish, and the 1 and 4 mm samples were collected from the excised macula before gentle dissociation in 20 units/ml of papain with 0.005% DNase I (Worthington Biochemical Corporation, Lakewood NJ, USA) on a shaker at 37°C for 1–1.25 h. The dissociated retinal samples were resuspended in DMSO-based Recovery Cell Culture Freezing Media (Life Technologies, Grand Island NY, USA) and frozen at −80°C in a CoolCell LX container (Corning NY, USA) to cool at 1°C/min. After samples were frozen for 3–12 h, they were transferred to liquid nitrogen for long-term storage.

Cryopreserved retinal samples were rapidly thawed in parallel at 37°C and resuspended in phosphatidylcholine buffer solution with 0.04% non-acetylated bovine serum albumin (New England Biolabs, Ipswich MA, USA) at a concentration of 1000 cells/μl. Single cells were then microfluidically separated and barcoded with the Chromium system v3.1 chemistry kit (10X Genomics, Pleasanton CA, USA). Resulting libraries were pooled and sequenced on the NovaSeq 6000 platform (Illumina, San Diego CA, USA), generating 100-base pair paired-end reads.

### Computational analysis of scRNA-seq data

FASTQ files were generated from base calls with the bcl2fastq software (Illumina, San Diego CA, USA). Next, FASTQ files were mapped to the pre-built GRCh38 reference with CellRanger (v3.0.1). Cells with fewer than 400 and greater than 6500 unique genes per cell were filtered, as were cells with >60% of genes mapping to the mitochondrial genome [to eliminate partially lysed cells from the dataset (56)]. Next, the filtered libraries were aggregated and normalized with canonical correlation analysis using the Seurat R package [v3.2.3; (57)]. Variable features were identified with the variance stabilizing transformation (vst) selection method.

Differential expression analysis was performed to identify genes enriched in foveal versus parafoveal libraries for each cell type. However, as differential expression analysis in Seurat can treat every cell as an independent observation, *P*-values may be inflated when comparing gene expression across a biological variable (such as foveal versus parafoveal region). Therefore for each celltype, we also perform a ‘pseudo-bulk’ differential expression by summing the counts for all reads in each independent library [e.g. biological replicate; (58)]. This pseudo-bulk comparison more accurately reflects the number of biological replicates between these retinal regions. Fully processed Single-cell RNA-sequencing data are hosted online at https://singlecell-eye.org for interactive visualization (43). Computational scripts for scRNA-seq analysis are available at github.com/drewvoigt10/RegionalRetina.

### Quantification of retinal cell densities

Cell density measurements were acquired from morphometric experiments by Curcio *et al.* (14,15) (https://christineacurcio.com/ResearchMaterial.html). A non-linear interpolation [as implemented in the R akima package (59)] was used to generate a smooth density map of cone photoreceptor cells, rod photoreceptor cells and RGCs.

### Immunohistochemistry

Immunohistochemistry experiments were performed on fixed frozen human tissue sections as previously described (26). Each section was blocked with 1 mg/ml of bovine serum albumin for 15 min before incubation with anti-FABP5 (1:50, Abcam, San Diego, CA, ab255276) primary antibody for 1 h. Sections were washed and incubated with Alexa-546-conjugated anti-rabbit IgG (1:200, Invitrogen, Carlsbad, CA) secondary antibody resuspended in PBS supplemented with DAPI (Sigma, St Louis, MO) for 30 min. Sections were washed and coverslipped before imaging alongside negative controls without primary antibody.

## Data Availability

The datasets and computer code produces in this study are available in the following databases:

- Raw and processed bulk RNA sequencing data have been deposited in GEO: GSE169046 (*URL embargoed prior to acceptance of this work*).
- Raw and processed scRNA-seq data have been deposited in GEO: GSE169047 (*URL embargoed prior to acceptance of this work*).
- The scRNA-seq data is hosted online at https://singlecell-eye.org for interactive visualization (43).
- All computational scripts for scRNA-seq analysis are available at github.com/drewvoigt10/RegionalRetina.

## Supplementary Material

SI_Figure_1-01_ddab140

SI_Figure_2-01_ddab140

SI_Figure_3-01_ddab140

SI_Table1_ddab140

SI_Table2_ddab140

SI_Table3_ddab140
