# Supplementary figures and images for: Human photoreceptor cells from different macular subregions have distinct transcriptional profiles
Source: Hum Mol Genet. 2021 May 20;30(16):1543–58. doi: 10.1093/hmg/ddab140 (PMC8330894; doi:10.1093/hmg/ddab140)

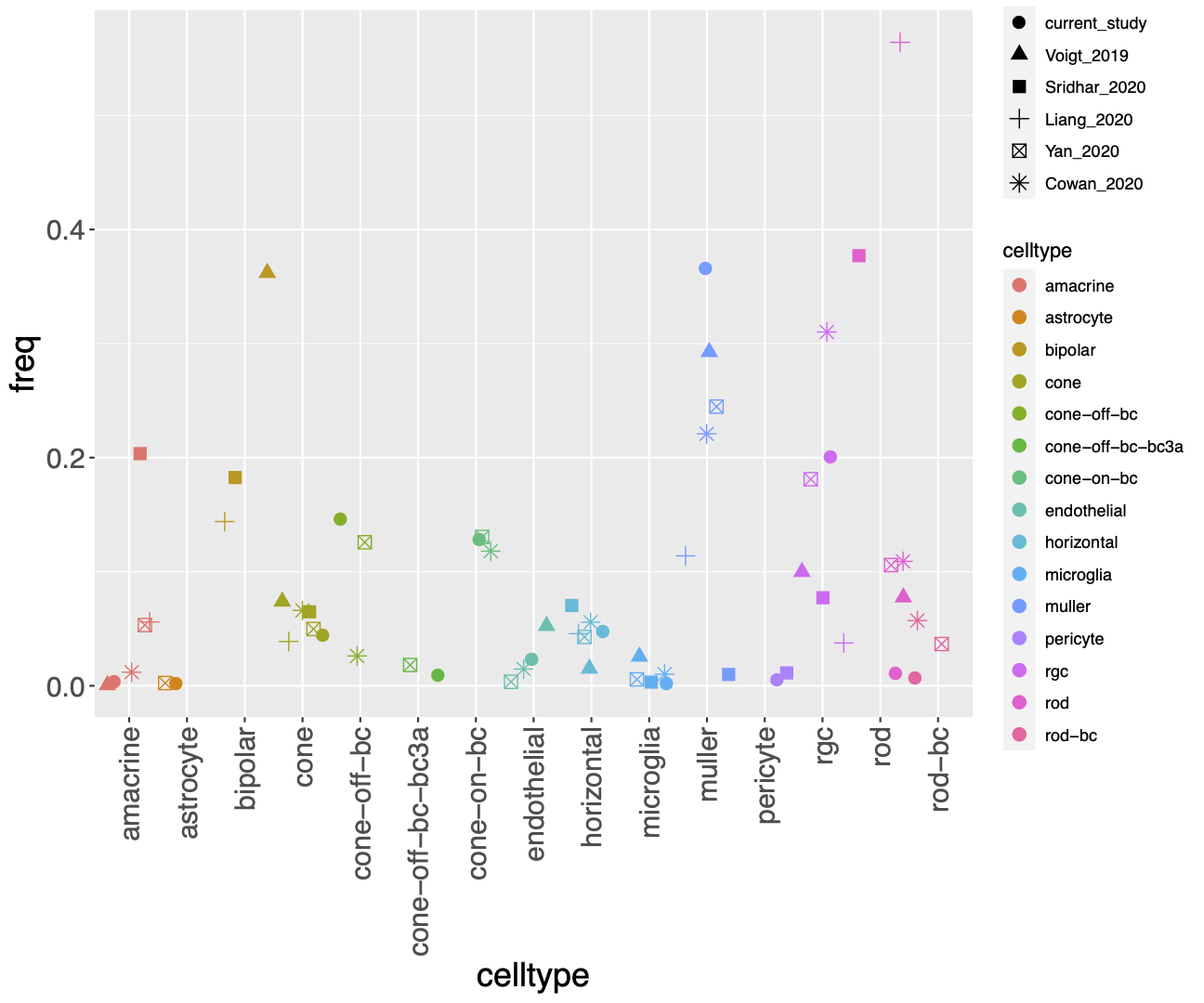

Supplement: SI_Figure_2-01_ddab140 [file si_figure_2-01_ddab140.zip › SI_Figure_2-01_ddab140.tif]
